# Evaluation of the topical gel and oral administration of *Punica Granatum Var Pleniflora* on oral mucositis induced by 5-Fluorouracil in golden hamsters

**DOI:** 10.1186/s12906-023-04053-1

**Published:** 2023-07-07

**Authors:** Seyede Pegah Hamidi, Omid Koohi-Hosseinabadi, Sepideh Khaksar, Ali Ghanbariasad, Amir Reza Dehghanian, Azizallah Dehghan, Zahra Haddadi, Roxana Gorgin, Mojtaba Farjam, Hiva Alipanah

**Affiliations:** 1 Student Research Committee, Fasa University of Medical Sciences, Fasa, Iran; 2 Central Research Laboratory, Shiraz University of Medical Sciences, Shiraz, Iran; 3 Department of Plant Sciences, Faculty of Biological Sciences, Alzahra University, Tehran, Iran; 4 Department of Medical Biotechnology, School of Advanced Technologies in Medicine, Fasa University of Medical Sciences, Fasa, Iran; 5 Noncommunicable Diseases Research Center, Fasa University of Medical Sciences, Fasa, Iran; 6 Surgical and Clinical Pathology, Shiraz University of Medical Sciences, Shiraz, Iran; 7 Department of Physiology, School of Medicine, Fasa University of Medical Sciences, Fasa, Iran

**Keywords:** 5-fluorouracil, Oral mucositis, *Punica Granatum Var Pleniflora*, Malondialdehyde, Myeloperoxidase

## Abstract

**Background:**

Oral mucositis (OM), an acute inflammation of the oral cavity, is a common complication in patients undergoing invasive myeloblastic chemotherapy or radiation therapy. 5-fluorouracil (5-FU) is one of the most effective therapeutic drugs, but one of the common side effects of 5-FU administration is OM. Unfortunately, no suitable treatment has been found, so far to control its side effects. Studies showed that herbal medicine like *Punica granatum var pleniflora* (PGP) has medicinal properties such as anti-inflammatory and antibacterial and can be an alternative for the treatment of fungal infection. Accordingly, we decided to investigate the therapeutic effect of *PGP* in the treatment of OM caused by 5-FU in golden hamsters.

**Methods:**

Sixty male golden hamsters were divided into six main group. Chemotherapy with 5-FU at dose of 60 mg/kg was performed at a ten-day duration. Then, cheek pouches of the hamsters were scratched with an 18-gauge sterile needle to induce oral mucositis in animals. On the twelfth day, as a day of intensification of OM, treatment with PGP including topical gel with concentrations of 5% and 10% and oral administration of hydro-alcoholic extract with doses of 125 mg/kg and 250 mg/kg for three- and five-day therapeutic duration were separately started. Finally, samples of cheek pouches in hamsters were collected on 14th and 17th days and histopathologic score (HPS), malondialdehyde (MDA), and myeloperoxidase (MPO) levels were assayed.

**Results:**

A significant (p < 0.05) decrease in histopathologic score was observed in G_10%−,_ P_125_-treated groups in comparison to the Ctrl group. Our data showed that treatment with G_10%_ is more potent than P_125_-treated group. In contrast, histopathologic score in G_10%,_ P_125_, and P_250_ treated groups demonstrated almost similar values On the 17th day. However, the levels of MDA and MPO in the treatment groups were enhanced compared with control group (p < 0.05).

**Conclusions:**

It is possible that PGP can play protective role in the healing of tissue damage caused by chemotherapy with 5-FU due to the presence of its natural compounds and antioxidant properties.

## Background

The incidence of cancer is constantly increasing in different societies and is known as the second leading cause of death in developed countries. However, head and neck cancers accounts for about 3–5% of all types of malignancies [1]. Most oral cancers are squamous cell carcinomas, and the median age at diagnosis is 60 years. Oral cancers most frequently affected the tongue, oropharynx, and floor of the mouth more than other sites such as lips, gums, dorsal surface of tongue, and soft palate [2]. 5-Fluorouracil (5-FU) is one of the most effective chemotherapy and anticancer drugs widely used in many types of cancer, including colon, breast, head, and neck cancers. However, it shows many side effects, including myelosuppression, dermatitis, and mucositis, during treatment [3].

Oral mucositis (OM) is an acute inflammation of the oral cavity caused by epithelial damage in patients who should be treated with chemotherapy drugs. This damage includes degenerative changes ranging from mild atrophy to severe ulcerations. Serious common and debilitating consequences include pain associated with opioid dependence, life-threatening infections, and prolonged hospitalization [4–6]. OM is a common complication in patients undergoing invasive myeloblastic chemotherapy or radiation therapy for cancers of the mouth, oropharynx, nasopharynx, or salivary glands [7, 8]. It is estimated that OM is seen in 40% of patients taking anticancer drugs, including 5-FU, which limits the use of high dose and reduces the effectiveness of treatment [9, 10]. So far, no standard method has been found to treat or prevent OM (8). Although the pain caused by OM can be cured with a series of interventions, but no suitable therapy has been found to treat OM [11].

In traditional Greek and Persian medicine, the flowers of the *Punica granatum var. pleniflora* (PGP) have benefits in the treatment of wounds, lung inflammation, diarrhea, digestive problems, infectious skin wounds, regeneration of male sexual organs, and diabetes [12, 13]. The topical use of PGP has been shown to be effective in controlling oral inflammation and reducing the accumulation of bacteria and fungi in periodontal diseases and dental stomatitis related to the most common oral cavity-colonising fungus, *Candida albicans*. A hydroalcoholic extract of this plant called “HAEP” has been reported to be effective against Staphylococcus, Streptococcus, Klebsiella, and proteozoa species, as well as *Escherichia coli* [14]. *Gavanji* et al. attempted to study the antimicrobial activity of three types of *punica granutum* extract, named *P. granatum var. pleniflora* (PGP), *P. granatum var. Sweet Alak, P. granatum var. Saveh* against *Candida albicans* and *Escherichia coli* in comparison with antibiotics. The results of this study showed that PGP had the highest antimicrobial effect and the highest content of phenolic compounds and antioxidants [15]. In the another study, the effects of PGP in inhibiting the bacterial growth of *Staphylococcus aureus* and *Escherichia coli* were shown [16]. Antibacterial, antioxidant, anti-inflammatory, and cytotoxic properties of PGP were also attributed to high percentage of phenolic compounds [17]. For example, treatment of intestinal epithelial cells with polyphenols from *Punica granatum L*., as noted by *Pepe et al.*., can reduce both inflammatory and oxidative stress parameters (cytokines release, cyclooxygenase-2) and apoptosis [18]. As well as, the study of PGP mouthwash on the treatment of gingivitis in diabetic patients (a clinical trial study) also revealed that PGP in comparison with chlorhexidine mouthwash had more efficacy in improvement of oral inflammation [19]. In addition, radioprotective effect of *Punica granatum* extract on radiation dermatitis and mucositis was investigated in a clinical, double blind, case control study on head and neck cancer patients [20]. In addition, in animal models, the therapeutic and protective effects of PGP on post-surgical peritoneal adhesions in rat model, aphthous stomatitis in mice model, and CCl4-induced hepatotoxicity in rat model have been demonstrated [21–23]. As well as, studies showed that PGP has medicinal properties such as anti-inflammatory and antibacterial and can be an alternative for the treatment of fungal infection [17, 24–27].

Topical gel treatment of skin diseases due to its direct use on the affected site, reduction of systemic side effects, and high acceptance from patients has become very attractive compared to parenteral or oral drug administration [28–30]. Accordingly, we decided to investigate PGP hydroalcoholic extract and PGP gel on reducing the side effects of the 5-FU in OM animal model due to its antioxidant and anti-inflammatory properties of PGP.

## Materials and methods

### Animals and group assignment

Sixty male golden hamsters with an average age of 6–8 weeks and a weight of 100 ± 20 gr were kept in standard conditions of 12:12 light–dark cycle at an ambient temperature of 22 ± 2 °C and 55% humidity. Food and water were available *ad libitum* for animals. The experiments were performed based on the Principles of Laboratory Animal Care by the Fasa University of Medical Sciences (Code: IR.FUMS.REC.1394.199).

### Experimental design

Animals were randomly divided into six groups including control, base gel–received group, topical treatment with 5% *Punica Granatum Var Pleniflora* (PGP) gel, topical treatment with 10% PGP gel, oral treatment with 125 mg/kg (≈ 1/6 LD_50_) hydroalcoholic extract, and oral treatment with 250 mg/kg (≈ 1/3 LD_50_) hydroalcoholic extract [31]. Control group (n = 10) received gastric gavage of distilled water. Treatment groups divided to two categories: (I) Topical treatment groups were divided into three subgroups, which received the base gel (G_b_) (n = 10), 5% gel (G_5_) (n = 10), and 10% gel (G_10_) (n = 10) once a day (in the morning), separately. Gels were administrated with a swab on both sides of cheek pouch and the wound area was completely covered with the gel. (II) Oral treatment groups were divided into two subgroups, which received gastric gavage of hydroalcoholic extract with doses of 125 mg/kg (P_125_) (n = 10) and 250 mg/kg (P_250_) (n = 10) once a day, separately. Oral mucositis model was performed on all groups. A 10-day period took to induce this model. Treatment on hamsters was carried out on 12th day (as day of intensification of OM). Five animals from control and treatment groups were randomly selected for three-day therapeutic duration and were sacrificed on 14th day (n = 5) and five animals from control and treatment groups were randomly selected for five-day therapeutic duration and were sacrificed and 17th day (n = 5) [32]. Excisional biopsies of the cheek pouch mucosa were performed. Eventually, histopathologic score (HPS), malondialdehyde (MDA), and myeloperoxidase (MDA) levels of samples were evaluated.

### Preparation of PGP hydroalcoholic extract

The PGP were prepared in Shiraz (Iran) and then its herbarium number were determined by the department of Traditional Medicine of Shiraz University of Medical Sciences (Index Herbarium code: *punica* PM 1375-*granatum* var. *pleniflora*) and transferred to the laboratory. Fresh flowers of PGP were cleaned and were dried at room temperature. 100 gr of PGP powder was placed in the percolator with 1000 ml of 70% ethanol for 72 h; then, the excess solvent was separated with a rotary to be completely concentrated. Finally, the extract was powdered through the processes of vacuum desiccator pump.

### Preparation of PGP gel

The carboxymethylation reaction was carried out to prepare of topical gel. Carboxymethyl cellulose 2% was used as the gel base. Two gr cellulosic material (textile powder) was weighed and was suspended in 98 ml of distilled water for 1 h to make a jelly state. Then, it was kept in 4 ^o^C for 24 h. For preparing topical gel, 5 and 10 gr of PGP dry extract were respectively added to base gel and 100 gr of final products were provided. Then, the container (100 gr) was covered with an aluminum foil and kept at 4 ^o^C for 24 h.

### Induction of oral mucositis model

Young male Golden *Syrian* hamsters (100 gr, 8 weeks old) received three intraperitoneal injections of a vial containing a solution of 5-FU at 50 mg/mL (Darou Darman Pars Company, Iran) with dose of 60 mg/kg [33] on zero, 5th, and 10th days. On 1th, 3th, and 4th days, cheek pouches of the hamster were scratched with an 18-gauge sterile needle to exert the chronic irritation [33–36].

### Measurement of body weight

Weight of animal as a physiological parameter was measured by a digital scale to evaluate the effects of 5-FU and PGP administration.

### Collecting tissue samples

**Fig. 1:**
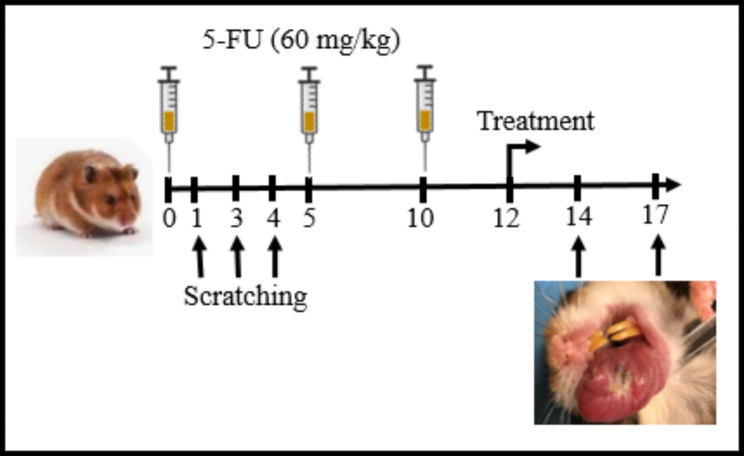
Experimental protocol

Treatments (topical and oral) on hamsters were carried out on 12th day. Animals were randomly sacrificed with ketamine (100 mg/kg) and xylazine (10 mg/kg) on 14th (n = 5) and 17th (n = 5) days to collect tissue samples. From each hamster, two samples were prepared. One sample from the right cheek to assay histopathological parameters and the other from the left cheek to evaluate MDA and MPO. Tissue samples were chosen from areas with mucosal involvement. A time-line was designed for this study (Fig. 1).

### Histopathological study

The tissue biopsies were fixed in formaldehyde and sections with 5 micrometer thickness were prepared from paraffin-embedded tissue blocks using a rotary microtome. The prepared slices were placed on a microscope slide and were stained with Hematoxylin & Eosin and after mounting, they were subjected to histological study by an Olympus light microscope (model BX51, Japan). The semi-quantitative analysis was performed as described by Lima et al. (2005) [37] (Table 1).

**Table 1:** Microscopically scoring system

Score	Description
0	normal epithelium, connective tissue without vascular dilatation, cell infiltration and absence of bleeding wound and abscess areas
1	observation of vascular congestion, infiltration of mild inflammatory cells with the predominance of mononuclear cells, absence of bleeding areas, edema, ulcer and abscess.
2	observation of moderate vascular congestion and areas of destruction of the epithelium, infiltration of inflammatory cells with the predominance of neutrophils, the presence of bleeding areas, edema and subsequent wound, but without the presence of an abscess.
3	observation of severe vascular congestion and dilatation of blood vessels, infiltration of inflammatory cells with the predominance of neutrophils, the presence of bleeding areas, severe edema, wound and abscess.

### Malondialdehyde (MDA) and myeloperoxidase (MPO) assay

MDA (as a lipid peroxidation product) and MPO levels (as a biomarker for.

inflammatory response) were evaluated using commercial colorimetrical assay Kit (Crystal day Biotech Co.). For MDA assay, tissue prepared from the left cheek of the hamsters was kept under liquid nitrogen. Buffer Lysis (300 µl) and BHT (butylated hydroxytoluene) (3 µl) were added to 10 mg of the desired tissue and then homogenized. Supernatant was collected and was centrifuged at 13,000 rpm for 3 min and its supernatant was used as a sample. For MPO assay, 10 mg of the sample was weighed and washed with cold PBS, then, it was homogenized using buffer lysis and was centrifuged at 10,000 rpm for 10 min. finally, supernatant liquid was used as a sample to measure MPO of samples.

### Statistical analysis

The data was analyzed using SPSS statistical program (SPSS v25.0 version). Kruskal-Wallis was used to compare the mean of the variables. Mann-Whitney test was performed for two-by-two comparison between groups. P value was corrected by Bonferroni correction. Data was displayed as Mean ± SD and p-values higher than 0.05 were not considered statistically significant.

## Results

### Measurement of body weight of animal

The weight of the animals during the study was measured by a digital scale. The results of body weight are listed in Table 2. There was no significant difference in the average of body weight between experimental groups.

**Table 2:** The average weight of the animals on the 14th and 17th days of the experimental period

Groups	P_250_		P_125_		G_b_		G_10_		G_5_		Ctrl	
**Day**	14	17	14	17	14	17	14	17	14	17	14	17
**mean**	99.40	127.20	117.66	106.23	116.60	95.33	106.00	11.80	119.75	120.00	106.40	117.20
**SD**	9.63	15.20	10.03	9.71	16.71	8.62	13.32	28.07	15.63	16.75	11.61	10.75

Ctrl: control group, G_b_: base gel group, G_5_: 5% PGP gel group, G_10_: 10% PGP gel group, P_125_: oral treatment with dose 125 mg/kg, P_250_: oral treatment with dose 250 mg/kg.

### Histopathological analysis

**Fig. 2:**
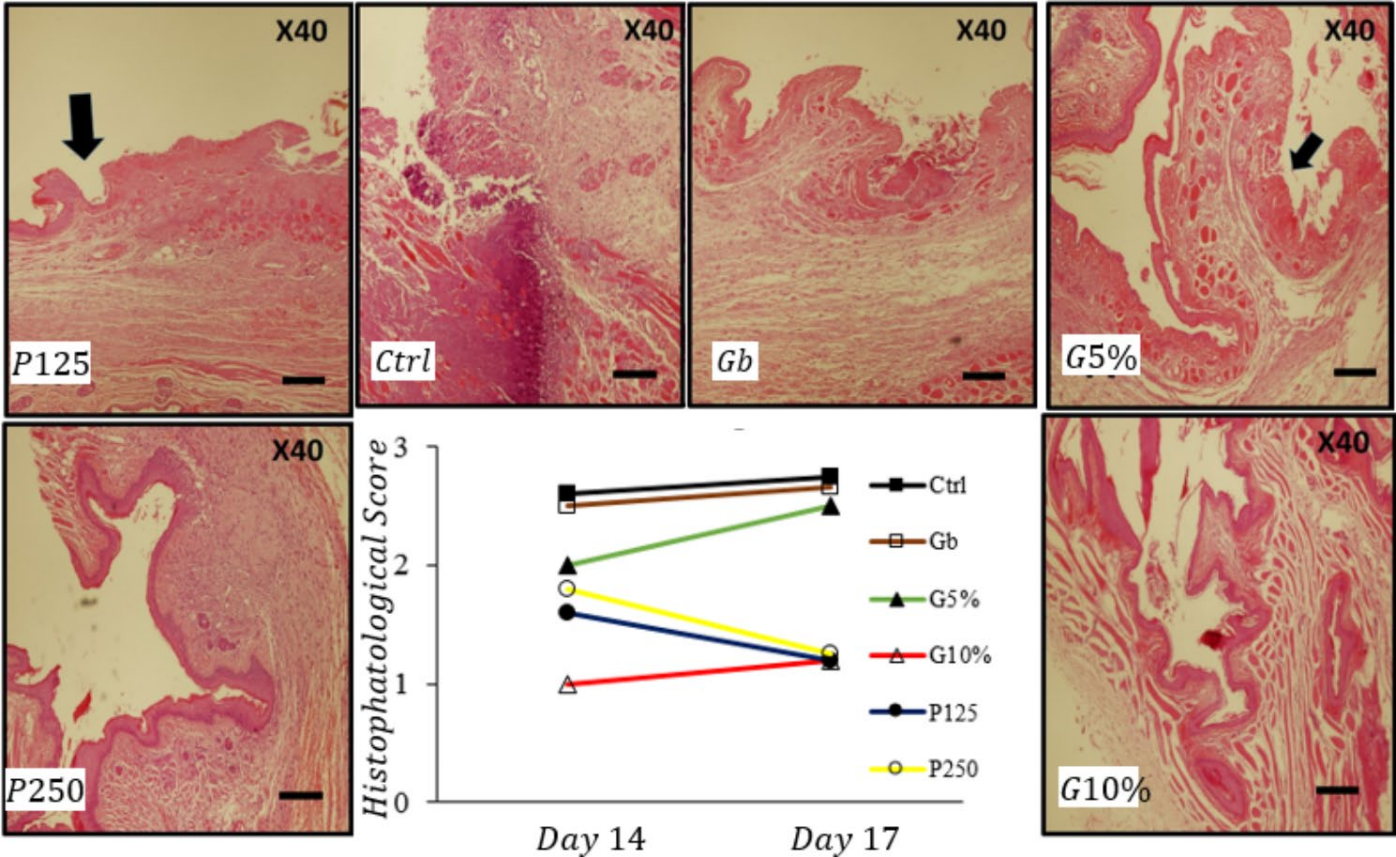
(I) Histomorphology of the hamster cheek pouch in experimental groups. (II) Graph show histopathologic score on 14th and 17th days. Exposure of rats with 5-FU significantly increased tissue damage. A significant (p < 0.05) decrease in histopathologic score was observed in G10%-, P125-treated groups in comparison to the Ctrl group. Results showed that treatment with G10% is more potent than P125-treated group. In contrast, histopathologic score in G10%, P125, and P250 treated groups demonstrated almost similar values on the 17th day. Values were expressed as the mean ± SD (n = 5). (Mann-Whitney U Test). Ctrl: control group, G_b_: base gel group, G_5_: 5% PGP gel group, G_10_: 10% PGP gel group, P_125_: oral treatment with dose 125 mg/kg, P_250_: oral treatment with dose 250 mg/kg. Scale bar for each micrograph is shown in the lower right corner as 800 μm

Severity of oral mucositis in tissues was evaluated accordance to a standardized scoring system of the histopathological analysis (Scoring: 0–3). This scoring was defined based on presence of inflammatory factors, wounds, abscesses, and inflammatory cell infiltration. On the 14th day, statistical analysis of histopathological results showed that tissue damage in the oral treatment group with dose 125 mg/kg was reduced (score: 0.89 ± 1.6; mild inflammation) compared to the control group (score: 2.6 ± 0.54) (P = 0.004) (Fig. 2). On the 17th day (five-day therapeutic duration), tissue damage in the oral treatment groups with dose of 125 mg/kg (score: 0.44 ± 1.20) was attenuated compared to the control group (score: 0.50 ± 2.75) (P = 0.009). In addition, statistical analysis of histopathological scoring between the gel base and control groups did not show any difference on both days. A significant decrease in histopathological scoring was observed in topical 10% gel group compared with gel base on the 17th day (P = 0.008) (Fig. 2).

### Evaluation of Malondialdehyde (MDA) concentration

Tissue MDA Level in experimental groups on 14th and 17th days was shown in Table 3. There were no significant differences between MDA Level in topical and oral treatment groups on 14th and 17th days. Unexpectedly, MDA level in topical treatment with 10% gel and oral treatment with doses of 125 and 250 mg/kg was significantly enhanced compared with control group on 14th day (*P* < 0.05, *P* < 0.05, *P* < 0.05, respectively). On 17th day, MDA concentration in groups treated with 5% gel and oral treatment with 250 mg/kg increased in comparison with control group (*P* < 0.05, *P* < 0.05, respectively). In general, both treatment protocol could not reduce the amount of MDA concentration and there was no difference in MDA levels on the 14th and 17th days (Table 3).

**Table 3:** Malondialdehyde (MDA) concentration in experimental groups

Groups	Ctrl	G_b_	G_5_	G_10_	P_125_	P_250_
Day 14th	2.48 ± 0.26	2.77 ± 0.15	3.17 ± 0.21	3.33 ± 0.24^*^	3.29 ± 0.26^*^	3.35 ± 0.12^*^
Day 17th	2.71 ± 0.29	2.70 ± 0.26	3.96 ± 0.53^*#^	3.29 ± 0.23	3.28 ± 0.31	3.44 ± 0.48^*^

Ctrl: control group, G_b_: base gel group, G_5_: 5% PGP gel group, G_10_: 10% PGP gel group, P_125_: oral treatment with dose 125 mg/kg, P_250_: oral treatment with dose 250 mg/kg.

*Significant difference between treatment and control groups (*P* < 0.05), #Significant difference between topical treatment and gel base groups (*P* < 0.05)

### Evaluation of myeloperoxidase (MPO) concentration

Tissue MPO Level in experimental groups on 14th and 17th days was shown in Table 4. The analysis of the results showed that the oral treatment with dose 250 mg/kg PGP extract increased the MPO level on both 14th and 17th days. In addition, Treatment with topical gel on both days could not cause a significant change in the MPO level compared to the control group.

**Table 4:** Myeloperoxidase (MPO) level in experimental groups

Groups	Ctrl	G_b_	G_5_	G_10_	P_125_	P_250_
Day 14th	3.16 ± 0.27	3.33 ± 0.17	4.04 ± 0.69	3.53 ± 0.28	3.41 ± 0.62	4.82 ± 0.65*
Day 17th	3.13 ± 0.21	3.31 ± 0.04	3.94 ± 0.31	3.58 ± 0.17	3.45 ± 0.33	4.86 ± 49*

Ctrl: control group, G_b_: base gel group, G_5_: 5% PGP gel group, G_10_: 10% PGP gel group, P_125_: oral treatment with dose 125 mg/kg, P_250_: oral treatment with dose 250 mg/kg.

*Significant difference between treatment and control groups (*P* < 0.05)

## Discussion

Recent research suggests that secondary mucosal injury during cancer treatment is so complex. It seems to be caused by a wide range of cellular and molecular events that not only affect the epithelium, but also affects the underlying stroma disruption. This point of view is an important step in understanding the biological and pathological complexities related to mucosal injury and will be particularly effective on mucositis prevention and treatment [38].

In our study, the preventive effect of the PGP extract on the oral mucositis injury caused by 5-FU was investigated. Results of histopathological scoring showed that topical gel and oral treatment were led to the improvement of tissue damage. Among the treatment groups, oral extract with dose 250 mg/kg and topical 10% gel had the best therapeutic effects. In addition, the results showed, at least on the 14th day, that treatment with topical gel was more potent than PGP-treated group. Topical gel can be best for local absorption since the gel is applied directly to a particular skin area. Also, topical gel is designed to target their therapeutic effect locally to damaged tissue while minimizing systemic exposure [39]. As well as, histopathologic score in G_10%_, P_125_, and P_250_ treated groups demonstrated almost similar values on the 17th day. The results exhibited that the both of MDA level and MPO activities increased in the treatment groups. Many studies have shown the effect of herbal components on reducing the pathological symptoms of OM. For example, the therapeutic effect of *Carum carvi L.* hydro-alcoholic extract on OM showed that the severity of histopathological damage was lower in the treatment group and the level of MDA and MPO decreased and increased, respectively [40]. In another study, the effects of oral extract and topical gel of *Hypericum perforatum* on OM were investigated. The results indicated the improvement of histopathological damage and the reduction of MDA levels in the treatment groups, especially in oral extract-received group [34]. It has also been shown that administration of *Zizyphus jujuba* oral extract and topical gel improved the pathological damages in OM. In addition, MDA and MPO levels in the treatment groups decreased and increased, respectively [41]. Moreover, it has been reported that *eucalyptus* extract also attenuated tissue damage in OM, and at the same time, MDA and MPO levels were lower and higher in treatment groups, respectively [42]. In another study, the effect of the *Elaeagnus angustifolia* hydro-alcoholic extract on OM caused by 5-FU was evaluated and it was observed that both oral extract and topical gel decreased the tissue damage [43]. The results of these studies clearly show that the medical agents, dosage, treatment technique (local or systemic), and magnitude and duration of exposure can affect the clinical efficacy and the response of antioxidant enzymes.

The cytopathology study of OM shows that radio-chemotherapy changes many biological processes such as the activity of antioxidant enzymes, apoptosis, inflammatory responses, cell signaling [44]. Previous studies showed that PGP’s major polyphenolics components, such as sellagic acid make it as a powerful antioxidant [45, 46]. In addition, the ability of PGP for inhibiting macrophage-mediated oxidation of LDL, matrix metalloproteinases (MMPs) expression, and pro-inflammatory enzymes provides the evidence that PGP may play important role in inflammatory diseases [47, 48]. *Marques et al.*, have explored the protective effect of PGP in the acute inflammation mice model and hypothesized that PGP leaves extract suppresses inflammatory mediators [49]. In addition, PGP, as noted by *Houstonab et al.*., showed a significant anti-inflammatory activity, which was confirmed by downregulation of cyclooxygenase-2 expression in an ex vivo model [50]. Previous experimental studies confirm the effect of antioxidant and inflammatory response in wound healing in OM, diabetic, etc. experimental models [44, 51, 52].

The therapeutic effect of PGP, in addition to its anti-inflammatory and antioxidant activity, can be attributed to its effect on apoptosis and cell signaling pathway. For example, *Khlood et al.*. by examining efficiency of PGP in rats exposed to the vancomycin, reported that hepatotoxicity and nephrotoxicity induced by vancomycin were blocked by PGP, which was confirmed by modulating apoptotic genes expression and inhibition of ROS signaling network [53]. Besides, *Li et al.*, investigated protective effects of PGF in RAW 264.7 cells and detected anti-inflammatory features such as inhibition p38 MAPK and NF-κB signaling pathways [54]. Based on previous studies, it can finally be suggested that PGP can accelerated wound healing in OM animal model by improving the status of antioxidant enzymes SOD and CAT, reducing lipid peroxidation, and modulating inflammatory cytokines.

Antibacterial property of PGP is probably another therapeutic mechanism in OM model, because deep OM ulcers are associated with colonization of oral bacteria. Investigation about antimicrobial activity of the PGP alcoholic extract on the bacterial strains showed that PGP is effective against Gram-positive and negative bacteria that cause food poisoning [55]. The PGP antibacterial effect is directly related to its phenolic and flavonoid content [56]. PGP antimicrobial activity confirms by the previous studies of Gosset-Erard et al. [57], Elshafie et al. [58], Hanani, et al. [59]., and *Cruz-Valenzuela et al.* [60]., who demonstrated that PGP inhibited the growth zone of bacteria.

### Limitations and strengths

Limitations of present study included mortality in hamsters and the development of secondary and unexpected infections, so we had to exclude hamsters with secondary infections. One of the strengths of this study is that for the first time, the effect of PGP was investigated in OM model.

## Conclusion

The present results demonstrated that both extract and topical gel of PGP could play a notable role in reducing the symptoms of tissue damage such as vascular congestion, infiltration, and the presence of inflammatory cells such as neutrophils in a 5-FU-induced OM model. It is possible that PGP can play protective role in the healing of tissue damage caused by chemotherapy with 5-FU due to the presence of its natural compounds and antioxidant properties. However, further investigation should be performed to clear its protective mechanisms.

## Data Availability

All data generated or analyzed during this study are available from the corresponding author upon reasonable request.

## Abbreviations

5-FU: 5-fluorouracil
G_10_: 10% gel
G_5_: 5% gel
G_b_: Base gel
HPS: Histopathologic score
MDA: Malondialdehyde
MPO: Myeloperoxidase
OM: Oral Mucositis
P125: Hydroalcoholic extract with doses of 125 mg/kg
P250: Hydroalcoholic extract with doses of 250 mg/kg
PGP: *Punica granatum var pleniflora*
