# Correction: Evaluation of the topical gel and oral administration of *Punica Granatum Var Pleniflora* on oral mucositis induced by 5-Fluorouracil in golden hamsters

**DOI:** 10.1186/s12906-023-04093-7

**Published:** 2023-07-19

**Authors:** Seyede Pegah Hamidi, Omid Koohi-Hosseinabadi, Sepideh Khaksar, Ali Ghanbariasad, Amir Reza Dehghanian, Azizallah Dehghan, Zahra Haddadi, Roxana Gorgin, Mojtaba Farjam, Hiva Alipanah

**Affiliations:** 1 Student Research Committee, Fasa University of Medical Sciences, Fasa, Iran; 2 Central Research Laboratory, Shiraz University of Medical Sciences, Shiraz, Iran; 3 Department of Plant Sciences, Faculty of Biological Sciences, Alzahra University, Tehran, Iran; 4 Department of Medical Biotechnology, School of Advanced Technologies in Medicine, Fasa University of Medical Sciences, Fasa, Iran; 5 Noncommunicable Diseases Research Center, Fasa University of Medical Sciences, Fasa, Iran; 6 Surgical and Clinical Pathology, Shiraz University of Medical Sciences, Shiraz, Iran; 7 Department of Physiology, School of Medicine, Fasa University of Medical Sciences, Fasa, Iran

**Correction: BMC Complement Med Ther 23, 225 (2023)**

10.1186/s12906-023-04053-1

Following publication of the original article [1], the authors reported an error affiliation.

Affiliation 5 should be corrected to 6 and vice versa.

The author group has been updated above and the original article has been corrected.
